# Supplementary material for: Exploring Matrix Effects on Binding Properties and Characterization of Cotinine Molecularly Imprinted Polymer on Paper-Based Scaffold
Source: Polymers (Basel). 2019 Mar 26;11(3):570. doi: 10.3390/polym11030570 (PMC6474114; doi:10.3390/polym11030570)
Supplement: Supplementary file 1 [file polymers-11-00570-s001.pdf]

polymers

MDPI

Supplementary data

Exploring matrix effects on binding properties and characterization of cotinine molecularly imprinted polymer on paper-based scaffold

Nutcha Larpant[1], Yaneenart Suwanwong[2], Somchai Boonpangrak[3] and Wanida Laiwattanapaisal[4,5*]

1 Graduated Program in Clinical Biochemistry and Molecular Medicine, Faculty of Allied Health Sciences, Chulalongkorn University, Thailand; Nutchalp.l@gmail.com
2 Department of Clinical Microscopy, Faculty of Allied Health Sciences, Chulalongkorn University, Thailand;
3 Center for Research and Innovation, Faculty of Medical Technology, Mahidol University, Nakhon Pathom, Thailand
4 Department of Clinical Chemistry, Faculty of Allied Health Sciences, Chulalongkorn University, Thailand
5 Electrochemistry and Optical Spectroscopy Center of Excellence, Chulalongkorn University, Thailand
* Correspondence: Wanida.L@chula.ac.th

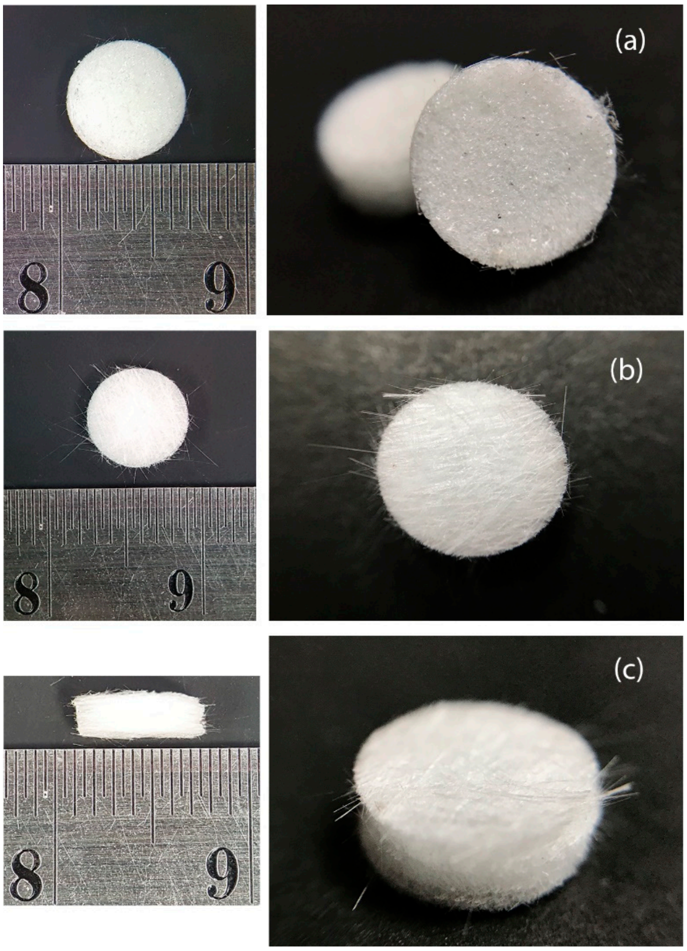

Figure S1. Microscopic pictures of glass fiber membranes. (**a**) MIP paper-based scaffold (front view). (**b**) Bare glass fiber membrane (front view). (**c**) Stack of four bare glass fiber membranes (side view).

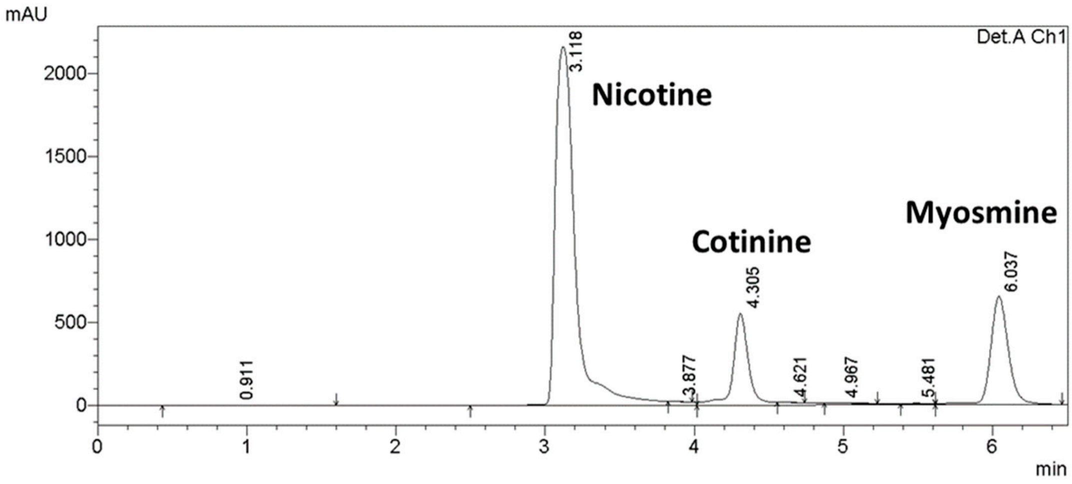

Figure S2. Chromatogram of standard mixture.

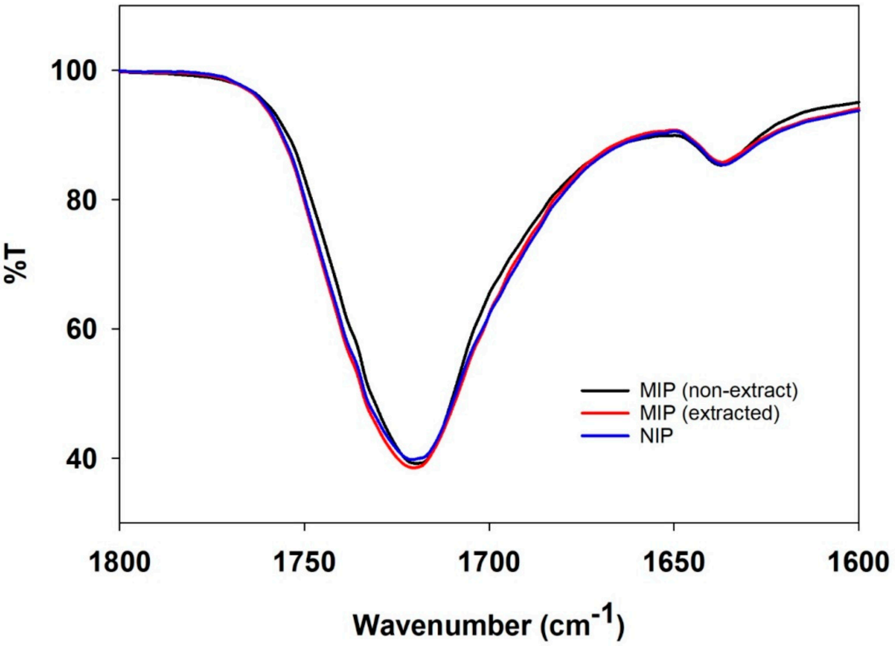

Figure S3. Infrared spectra of synthesized polymers. Red line represents MIP (nonextracted), blue line represents MIP (extracted), and black line represents NIP (focusing on 1720 cm^{-1}).

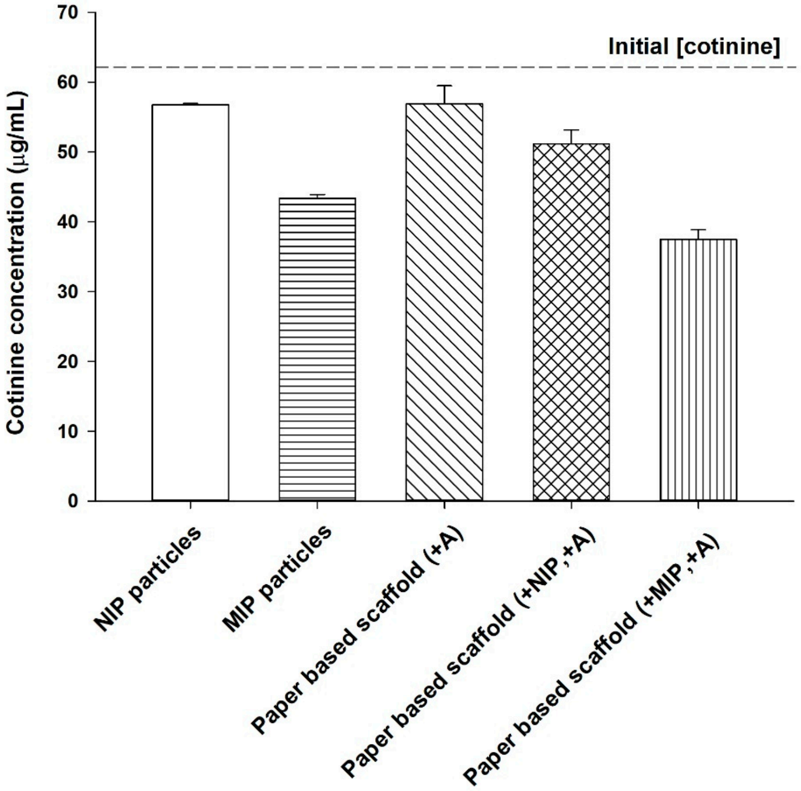

*+A; with agarose gel, +MIP; with MIP particles, +NIP; with NIP particles

Figure S4. Remaining cotinine standard after rebinding with different adsorbent materials. Black bar graph represents concentration of cotinine standard before rebinding.

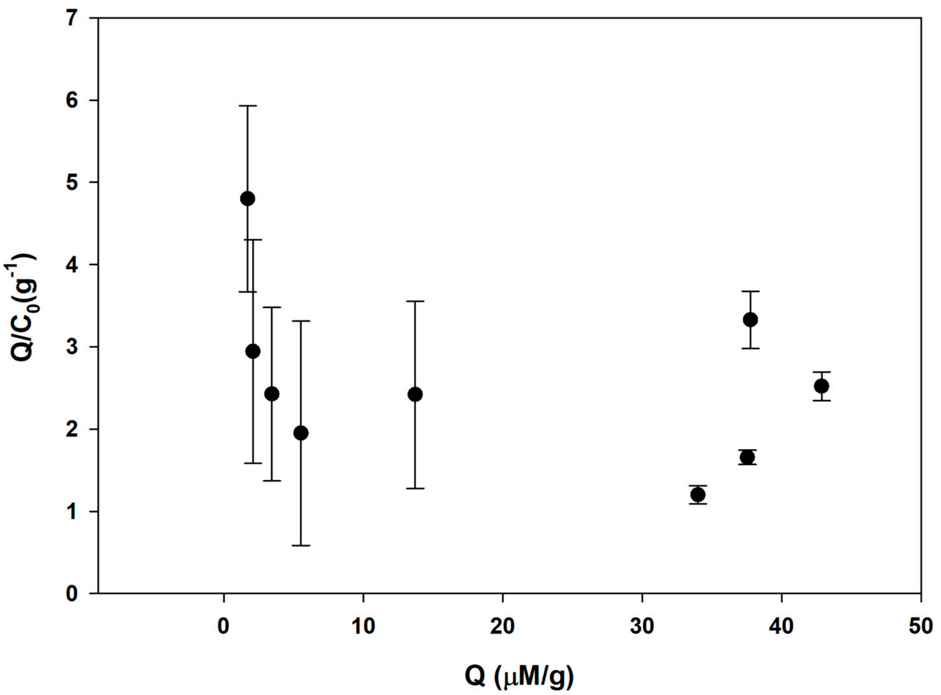

Figure S5. Scatchard plot of NIP.

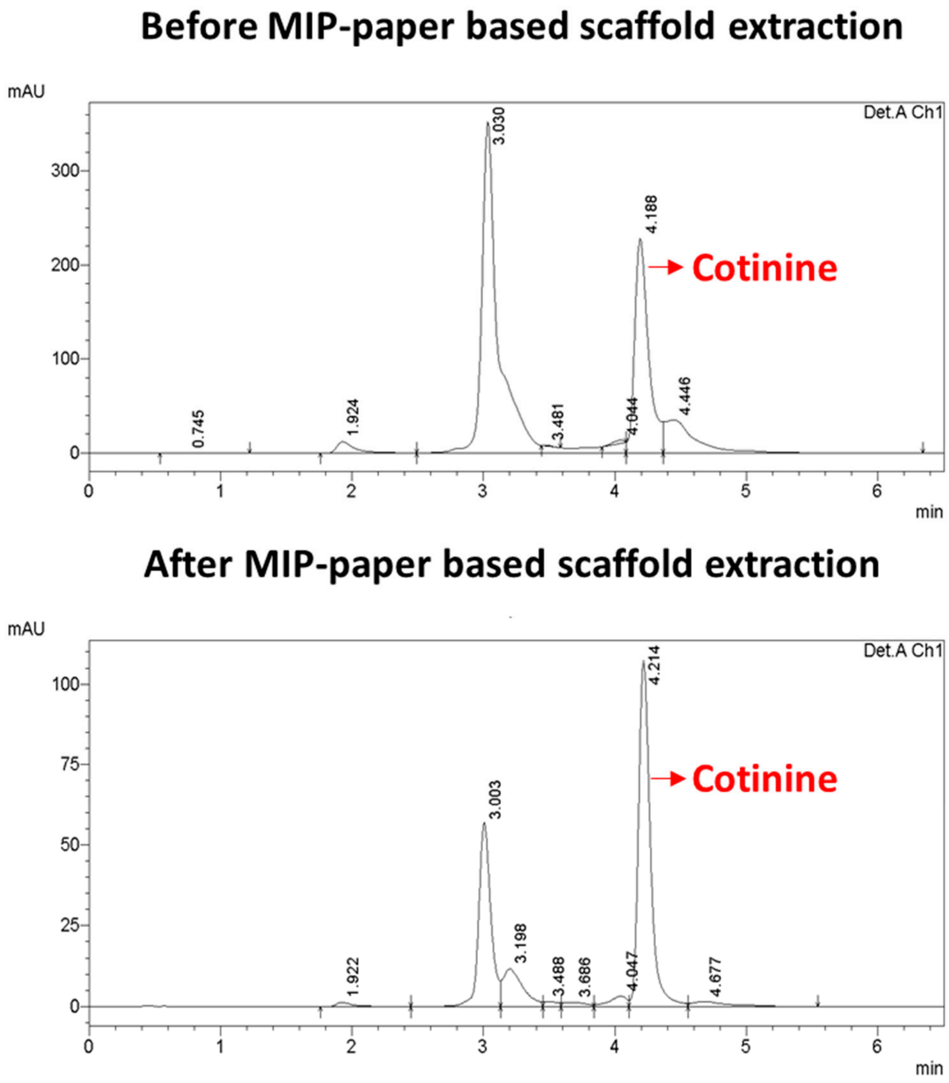

Figure S6. Chromatogram of eluted cotinine from MIP-paper based scaffold.

Table S1. Chromatographic parameters for HPLC method.

	**Nicotine**	**Cotinine**	**Myosmine**
Theoretical plate (*N*)	2480	11,142	9190
Capacity factor (k′)	2.42	3.72	5.65
Selectivity (α)[a]	-	1.54	1.52
Resolution (R_s)[a]	-	5.27	6.22
HETP (μm)	111	22	27

[a] With respect to previous peak.
HETP, height equivalent to theoretical plate.

Table S2. Concentration of adsorbed cotinine (μg/mL) from MIP paper-based scaffold and bare paper-based scaffold (initial concentration of cotinine: 5 μg/ mL).

Absorbent	Concentration of adsorbed cotinine (μg/mL)
Paper-based scaffold (with agarose; with MIPs)	3.8 ± 0.0810
Paper-based scaffold (with agarose; without MIPs)	0.63 ± 0.0042
